# An assessment of the provision of women-friendly care and its associated factors among mothers who gave birth at public health institutions in South Gondar Zone, Northwest Ethiopia

**DOI:** 10.3389/fgwh.2024.1368388

**Published:** 2024-05-27

**Authors:** Wassie Yazie Ferede, Besfat Berihun Erega, Enyew Dagnew Yehuala, Tigist Seid Yimer

**Affiliations:** Department of Midwifery, College of Health Sciences, Debre Tabor University, Debre Tabor, Ethiopia

**Keywords:** women, friendly care, childbirth, public health institutions, South Gondar

## Abstract

**Introduction:**

“Women-friendly care” is one of the categories of respectful maternal care and is a method of providing care that improves women's access to safe parenting and to reproductive health services by creating a friendly environment at all levels. Improving service use is crucial, particularly in situations where it is low. There is limited data on women-friendly care during childbirth in Ethiopia.

**Objective:**

This study aimed to assess the provision of women-friendly care and its associated factors among mothers who gave birth at health institutions in the South Gondar zone, Northwest Ethiopia.

**Methods:**

A multicenter institutional-based cross-sectional study design was conducted among mothers who gave birth at South Gondar Zone public health institutions, from February 01 to March 30/2021. Three hundred forty-eight study participants were selected by using systematic random sampling. A validated questionnaire was used for data collection. For analysis, the data were imported into Epi-Data version 4.6 and exported to SPSS version 25. A multivariable logistic regression analysis was performed to identify factors associated with the outcome variable. An adjusted odds ratio with a 95% confidence interval was computed to determine the level of significance.

**Results:**

The study had 344 participants in total, with a response rate of 98.85%. The study revealed that a full 73% [95%; CI: 68.6, 77.3%] its participants received women-friendly care. Having antenatal care follow-up [AOR: 3.02, 95% CI: 2.16–11.68], being a primipara [AOR = 2.30 95% CI: 1.23–5.49], not experiencing complications during childbirth [AOR: 2.13, 95% CI: 1.17–12.4], stays at health care facilities, specifically between 13 and 24 h [AOR: 0.25, 95% CI: 0.09–0.67], place for delivery [AOR: 2.01, 95% CI: 1.29–6.09] and delivering during daytime hours [AOR = 2.17, 95% CI: 1.08–5.65] were significantly associated with the provision of women-friendly care.

**Conclusions:**

Only two-thirds of the study participants received Women's-friendly care during childbirth. It was found to be low in our study area in contrast with the majority of the previous findings. Our own findings also suggest the importance of minimizing elective induction of labor during night, of providing comprehensive counseling on antenatal care follow-up, of ensuring mothers remain at health care facilities until the recommended duration, and of implementing early prevention and management of childbirth complications to ensure that mothers receive women-friendly care.

## 1 Introduction

“Women-friendly care” constitutes one of the categories of respectful maternal care and is a method of providing care that improves women's access to safe parenting and to reproductive health care services by creating an environment that is friendly at all levels. Improving the utilization of services is crucial, particularly in environments where it is low (1, 2). Mother-friendly childbirth practices are natural and healthy practices that are better for mothers, babies, and families. These practices are ways in which healthcare professionals can ensure that mothers and their families have a pleasant, healthy childbirth experience (3). This approach to maternity care focuses on the right of women to have access to and obtain quality care, which in turn has health benefits for their infants. Therefore, this approach is part of a broader strategy for reducing maternal and neonatal morbidity and mortality and requires strong partnerships between governments, health care systems, and communities (2).

According to a report on women's friendly health service experiences in maternal care, to be defined as providing “women-friendly” care, healthcare services must be located close to where women can easily access them, are little in cost, promote the care providers, and offer chances for clients to make informed decision-making concerning the care they receive (1). In 2020 an estimated 287,000 women worldwide died from a maternity-related cause that might have been easily avoided; in that same year, Sub-Saharan Africa alone accounted for approximately 70% of global maternal deaths, followed by Central and Southern Asia, which accounted for almost 17% of maternal deaths (4). The primary causes of the poor quality of women's healthcare services are an inadequate infrastructure, a lack of supplies, a failure to detect and treat complications or emergency cases, a failure to comply with established standards, and inadequate client–provider communications (3).

A lack of “women-friendliness” often has greater impacts on the health of poor women who are at greater risk of maternal mortality and morbidity. Poor or illiterate women have less of a voice and may be more vulnerable to neglect, abuse, or miscommunication, especially when staff are stressed, overworked, or under too much pressure to be able to provide women-friendly care. Women who are disempowered cannot request quality health care nor can they demand accountability when the services provided are questionable (3).

A woman's relationships with her healthcare providers and with the maternity care system during pregnancy and childbirth is vitally important. Not only are these encounters the vehicles for essential and potential lifesaving healthcare services, but also women's experiences with their caregivers can have the impact of empower and comfort them or can inflict lasting damage and emotional traumas, which can add to or detract from women's confidence and self-esteem. A woman's memories of her childbearing experiences will persist for a lifetime, and she is often shares them with other women, thereby contributing to a climate of confidence or doubt about childbearing (4). Healthcare providers need to be able to understand women's needs and to recognize the uniqueness of the birthing experience for each woman and her family, including providing care that is culturally sensitive and women friendly (5). The presence of women-friendly care in a healthcare facility can determine labor outcomes for mothers as well as for babies. The existence of a clinically and administratively sound healthcare provision system does not necessarily ensure the utilization of healthcare services if the mother is dissatisfied with how the care is provided (1). In Germany, patients reported a lack of involvement in the planning of the healthcare process and said that they felt they were not being taken seriously. These patients also felt that their diagnoses had not been disclosed empathetically and that they were insufficiently informed about their disease (6). A study on Zambian women's experiences with urban maternity care indicated that despite 89% of the women reporting good care, 21% reported remembering someone who had treated them badly by shouting at or scolding them during labor, and one-fifth reported having been left alone in the labor room (7). A study conducted at a healthcare centre in Malawi, that assessed the quality of care and its impacts on the utilization of primary-level maternity services revealed high levels of satisfaction among patients regarding providers' attitudes (97%), technical competence (86%), and working hours (91%). Nevertheless, the study participants expressed dissatisfaction regarding the absence of privacy (8). We have found that, for most Ethiopian women, pregnancy entails not only the prospect of childbirth but also concerns for the well-being of both the woman and her unborn child. Consequently, during labor, many women prefer to be in an environment where they feel secure, appreciated, and capable of receiving emotional and practical assistance from both family members and healthcare providers (4). The development of more women-friendly healthcare facilities is part of a greater effort to ensure that all women with complications or emergencies can reach the right healthcare facility and can receive appropriate and timely services and care. Even if these services are technically competent or cost effective, they can be impersonal and inappropriate to the needs of women and their families (9).

According to the 2016 Ethiopia Demographic and Health Survey (EDHS) report, Ethiopia has one of the highest maternal mortality ratios (MMRs) globally, with 412 maternal deaths occurring per 100,000 live births (10). A negatively perceived quality of care in facility-based childbirth often prevents the use of facility-based delivery services, both in the present or in the future (11). According to a recent evaluation conducted in Ethiopia, 85% of the 103 surveyed women reported that their service experience had impacted their decision regarding their future place of delivery. Providing women-friendly care is a key intervention for improving care quality and for bringing previously unreached women to healthcare facilities where they can receive high quality for maternity care; according to Kene et al., full attention has not been given to the level of women-friendly care provision or to its associated factors (12). A few previous studies conducted in Ethiopia, including those in the Bale Zone in Southeast Ethiopia and in the Jimma Medical Center, have focused on the overall framework of women-friendly care and have revealed that a significant number of mothers are not cared for in a friendly manner, as 39% and 29%, respectively, did not receive women's friendly care (12, 13). To improve the quality of health care and the ability of healthcare providers to respond to women's needs, this study aimed to evaluate the level of women-friendly care provision and to identify associated factors among mothers during the immediate postdelivery period at public hospitals in southeast Ethiopia.

## 2 Methods

### 2.1 Study design, period, and research area

We conducted a study that we designed to assess the degree to which how women receive friendly care during labor and delivery in public healthcare facilities across the South Gondar Zone in the Amhara region of Northwest Ethiopia. Our study took place from February 1 to March 30, 2021, and, for this study, we used a multicenter institution-based cross-sectional study design. The South Gondar Zone, with a population of approximately 2.6 million people, has a mix of rural and urban areas, and consists of 18 woredas (districts). Healthcare services are provided through a network of 403 health posts, 96 public health centres, 140 private clinics, and 9 governmental hospitals, all of which continuously offer delivery services.

### 2.2 Source population and study population

Our source population consisted of mothers who gave birth at the South Gondar Zone Public Health Institutions and who were chosen from public healthcare institutions during the study period and were selected by systematic random sampling.

### 2.3 Sample size and sampling procedure

We calculated our sample size by using the single population proportion formula while considering assumptions such as a 95% confidence interval, a 5% margin of error, and The magnitude of women-friendly care and its associated factors among mothers during childbirth in the Jimma zone, southern Ethiopia, was found to be 71% (*p* = 0.71) according to Hurissa et al. (14).

$$n\;=\;{\left(Za/2\right)}^{2}{{\;}_{\times \;P\;\left(1-P\right)/d}}^{2}n\;=\;{\left(1.96\right)}^{2}\;\times \;0.71\;\left(1.0-0.71\right)/0.05^{2}=\;316$$

After considering a 10% nonresponse rate added to the sample size calculated above, the total sample size was found to be 348.

We used a simple random sampling technique to select the Debre Tabor Comprehensive Specialized Hospital (154), the Adiss Zemen Primary Hospitals (46), the Nefasmewucha Primary Hospitals (36), and 20 healthcare centers (112). The sample was allocated proportionally to all selected public health institutions based on the two-monthly average number of women who gave birth at each institution in 2020.

We utilized a systematic random sampling technique to select study participants from mothers who gave birth at public healthcare institutions in the South Gondar zone until the required sample size was obtained. The data were collected from every third woman who gave birth during the study period at each selected healthcare institution. The sampling interval *k* = 3 was calculated by dividing the source population by the total sample size, and this interval was used in all health institutions to select study participants. For each of the public health institutions, the constant number *K* was also calculated, and it was the same at *K* = 3. We used this interval in all public healthcare institutions to select study participants. The first sample was selected randomly by the lottery method from among the first three participants (one randomly selected); then, every 3rd unit was taken to obtain the required sample size from each institution.

### 2.4 Data collection tools and procedure

We utilized a validated quantitative data collection tool, for the outcome variable, with a Cronbach's alpha of 0.889 (15), and we developed the factors affecting the women- friendly care Questionnaire by reviewing various studies (10–19). We also conducted face-to-face interviews with participants were also conducted to collect our data.

The questionnaire was initially written in English, then was translated into Amharic (which is the primary language of our study participants), and finally was back-translated into English to ensure uniformity. We divided our tool/questionnaire into four sections: the first section include the sociodemographic information of the respondents; the second section is a questionnaire with information on the participants' obstetric characteristics; the third section includes provider-related factors; and the fourth section include the categories of women- friendly care that women received during facility-based childbirth. To aid in the data collection procedure, we engaged twenty data collectors with BScs in midwifery and three MSc midwifery specialists' supervisors. A postpartum exit interview was conducted with women who had just given birth in these facilities. After we checked over the participants completed the questionnaires for any missing items and for accuracy, they were collected and signed by their MSc midwifery specialists' supervisors. In addition, there was continuous follow-up and supervision by the principal investigator throughout the data collection period.

A half-day training was provided for the data collectors and supervisors about the purpose of the study and the techniques of data collection. The trained data collectors were supervised during the data collection. A pretest (20) was performed on 5% of the sample (18 women) before the actual data were collected at Ebnat Primary Hospital and at Wogeda Health Center. The collected data were reviewed and checked for completeness, clarity, and consistency, and on-spot corrective measures were taken by both the data collectors and their supervisors.

### 2.5 Data processing and analysis

After the data were cleaned for errors and missing values, they were input into Epi-Data version 4.6 and exported to SPSS version 23 for analysis. Analytical and descriptive methods of statistical analysis were employed. To determine how each independent variable is associated with the outcome variable, bivariable and multivariable logistic regression were used. Finally, all independent variables with binary logistic regression *p* ≤ 0.25 were entered into multivariable logistic regression for further analysis, and significant associations were identified based on *p* < 0.05 and adjusted odds ratios (AORs) with 95% CIs.

The results were compiled and are presented in tables, graphs, and text, and the results were evaluated using odds ratios (ORs) and 95% confidence intervals (CIs).

### 2.6 Operational definition of variables

#### 2.6.1 Women's friendly care

Care that is provided with in a kind and friendly approach to women, using polite words, calling the woman by her name, and speaking the language the mother understands, it is measured by using seven requirements. A woman who answered “Yes” to all seven questions regarding friendly care was considered to have received it during labor and delivery (11, 12).

## 3 Results

### 3.1 Sociodemographic characteristics of the study participants

To recap from the above, we note here that a total of 344 women participated in this study, for a response rate of 98.85%. Among the participants, 36.9% were aged 25–29 years, with a median age of 27 years and an interquartile range of (IQ 22–30) years. Of the total respondents, 323 (93.9%) were Amhara by ethnicity, and 269 (78.2%) were Orthodox Christians. Regarding the marital status of the study participants, 310 (90.1%) were married, and 118 (34.3%) were housewives. Of the total respondents, 190 (55.2%) had a monthly family income <2,282 Ethiopian birrs (around 40 USD and 37 Euros) and 186 (54.1%) were rural residents (See Table 1).

**Table 1:** Mothers who gave birth at public healthcare institutions in the Southern Gondar Zone, Amhara region, Northwestern Ethiopia, 2021 and their sociodemographic characteristics (*n* = 344).

Characteristics	Categories	Frequency	Percentage
Age in groups	15–19	27	7.8
	20–24	80	23.3
	25–29	127	36.9
	30–34	60	17.5
	35 and above	50	14.5
Place of residence	Rural	186	54.1
	Urban	155	45.9
Religion	Orthodox	269	78.2
	Muslim	64	18.6
	Protestant	11	3.2
Marital status	Single	26	7.6
	Married	310	90.1
	Others(a)	8	2.3
Ethnicity	Amhara	323	93.9
	Tigre	11	3.2
	Other Ethnicity(b)	10	2.9
Educational status	Unable to read & write	93	27
	Able to read & write	58	16.9
	Primary (1–8)	25	7.3
	Secondary (9–12)	85	24.7
	Collage and above	83	24.1
Mothers occupation	Housewife	118	34.3
	Private employee’	41	11.9
	Government employee	48	14
	Merchant	54	15.7
	Student	37	10.8
	Farmer	46	13.4
Average monthly income	Less than 2,282	190	55.2
	More than 2,282	154	44.8

Keys: a = divorced and widowed, b = Oromo and Gurage.

### 3.2 Obstetric related characteristics of the study participants

The results of this study indicated that the majority of the respondents (241; 70%) were multiparous and that 311 (90.4%) had ANC follow-up data. Of the total respondents, 262 (76.2%) gave birth via spontaneous vaginal delivery, and 32 (9.3%) delivered via caesarean section. Of the total respondents, 322 (93.6%) gave birth to live babies, and 268 (77.9%) participants were attended by midwife healthcare professionals. Out of the total number of our study participants, 96, approximately 27.9% of these participants, did not have companions present during delivery, and 277 (80.5%) had a 12-h or less total stay at the healthcare institutions where they had given birth (Table 2).

**Table 2:** The obstetric characteristics of mothers who gave birth at public healthcare institutions in the Southern Gondar Zone, Amhara region, Northwest Ethiopia 2021 were recorded (*n* = 344).

Characteristics	Categories	Frequency	Percentage
Number of parity	Primi-para	103	30
	2–3	159	46.2
	≥4	82	23.8
pregnancy intended/wanted	Yes	296	86
	No	48	14
Antenatal care follow-ups	Yes	311	90.4
	No	33	9.6
Place of antenatal care follow-ups	Health center	203	65.3
	Primary hospital	26	8.3
	Referral hospital	55	17.7
	Private clinic	27	8.7
Number of ANC follow-ups	Once to twice	91	29.3
	Three times	133	42.7
	Four and above	87	28
Current place of delivery	Public health center	110	32
	Primary hospital	80	23.3
	Public referral Hospital	154	44.7
Provider conducting delivery	Nurse	2	0.6
	Midwife	268	77.9
	Doctor	25	7.3
	Emergency surgeon	49	14.2
Sex of provider	Male	201	58.4
	Female	143	41.6
Mode of delivery	SVD	262	76.2
	Cesarean section	32	9.3
	AVD	50	14.5
Delivery outcomes	Alive	322	93.6
	Stillbirth	22	6.4
Complications during labor and delivery	No	306	89
	Yes	38	11
Type of complication	Hemorrhage	11	3.2
	Hypertensive disorders	12	3.5
	Other complication©	15	4.4
Time of delivery	Day time	167	48.5
	Nighttime	177	51.5
Length of stay at health facilities	12 h or less	277	80.5
	13–24 h	24	7
	25 h and above	43	12.5
Companions during delivery	Yes	248	72.1
	No	96	27.9

SVD, spontaneous vaginal delivery; AVD, assisted vaginal delivery; ©=infection, obstructed labor, premature rupture of membrane.

### 3.3 The status of women- friendly care

**Figure 1:**
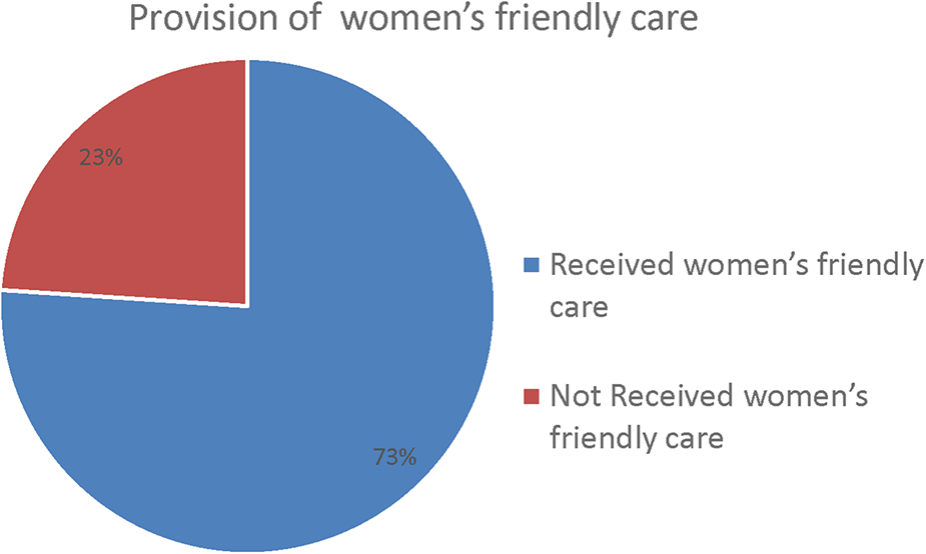
The provision of women-friendly care during childbirth.

Out of the 344 participants interviewed, a full 73% (95% CI: 68.6, 77.3%) of these women reported having received friendly care, but a significant number of women (27%) stated that they had not received friendly care during childbirth [95% CI: 22.7–31.4]. Of the participants, 27 (7.8%) did not receive information about pain relief measures, and 73 (21.2%) complained that healthcare professionals did not call them by their names (Table 3, Figure 1).

**Table 3:** Types of women-friendly care reported by mothers during childbirth at public health institutions in the Southern Gondar Zone, Amhara region, Northwest Ethiopia, 2021 (*N* = 344).

Category of RMC	Types of women friendly care	Yes%	No%
Friendly care	I felt that health workers cared for me with a kind approach	319 (92.7%)	25 (7.3%)
	The HWs treated me in a friendly manner	316 (91.9%)	28 (8.1%)
	The health workers were talking positively about pain and relief	317 (92.2%)	27 (7.8%)
	The health workers showed his/her concern and empathy	322 (93.6%)	22 (6.4%)
	All HWs treated me with respect as an individual	315 (91.6%)	29 (8.4%)
	The HWs speak to me in a language that I can understand	316 (91.9%)	28 (8.1%)
	The health provider called me by my name	271 (78.8%)	73 (21.2%)

HW, health care workers.

### 3.4 Factors associated with women's friendly care during childbirth

The results of the bivariable analysis showed that the respondent's ANC visit, parity, time of delivery, mode of delivery, place of delivery, total stay at the health facility, and complications during childbirth were factors that were found to have a *p* value of 0.25 or less and were candidates for the multivariable logistic regression model. In the multivariable logistic regression analysis, six variables, ANC visits, number of parities, time of delivery, total length of stay at the healthcare facility, place of delivery, and complications during childbirth, were found to be significantly associated with women-friendly care at a *P*-value of <0.05.

This study revealed that its participants who gave birth at a local healthcare center [AOR: 2.01, 95% CI: 1.29–6.09] were more than twice as likely to receive friendly care than those who delivered at a referral hospital.

This study also revealed that primiparous women (women who had given birth for the first time) were 2.3 (AOR = 2.30 95% CI: 1.23–5.49) times more likely to receive friendly care than women with a parity of four or more.

We also found that the study participants who had attended antenatal care (ANC) appointments were more than three (AOR: 3.02, 95% CI: 2.16–11.68) times more likely to receive friendly care than those who had not. Compared with those who delivered at night, women who delivered during the day were more than twice (AOR = 2.17, 95% CI = 1.08–5.65) as likely to receive friendly care.

This study revealed that the participants whose with total hours of stay at healthcare facilities were from 13 to 24 h were 75% less likely to receive friendly care [AOR: 0.25, 95% CI: 0.09–0.67] than those whose women with total hours of stay at healthcare facilities were 12 h or less.

This study also revealed that among those participants who did not experience complications during labor or delivery, were 2.13 (AOR: 2.13, 95% CI: 1.29–7.58) times more likely to receive women-friendly care than those who had faced complications during labor and delivery (Table 4).

**Table 4:** Factors associated with women friendly care during labor and childbirth at public health institutions in the South Gondar Zone, Amhara region, Northwest Ethiopia, 2021 (*N* = 344).

Variables	Received WFC service		COR (95% CI)	AOR (95% CI)
	No	Yes		
ANC visit				
No	15 (45.5%)	18 (54.5%)	1	1
Yes	236 (75.9%)	75 (24.1%)	3.77 (1.81–7.85)	3.02 (2.16–11.68)**
Parity				
Primi-para	80 (77.7%)	23 (22.3%)	2.46 (1.30–4.66)	2.30 (1.23–5.49)*
2–3	123 (77.4%)	36 (22.6%)	2.42 (1.36–4.30)	2.28 (1.17–4.40)
≥4	48 (58.5%)	34 (41.5%)	1	1
Time of delivery				
Day time	138 (82.6%)	29 (17.4%)	2.69 (1.62–4.46)	2.17 (1.08–5.65)*
Night time	113 (63.8%)	64 (36.2%)	1	1
Mode of delivery				
SVD	202 (77.1%)	60 (22.9%)	2.43 (1.29–4.58)	1.65 (0.74–3.50)
Cesarean section	20 (62.5%)	12 (37.5%)	1.20 (0.48–2.99)	0.36 (0.08–1.64)
AVD	29 (58%)	21 (42%)	1	1
Place of delivery				
Health center	92 (83.6%)	18 (16.4%)	2.45 (1.33–4.51)	2.01 (1.29–6.09)*
Primary hospital	55 (68.8%)	25 (31.3%)	1.05 (0.59–1.89)	1.00 (0.50–1.96)
Referral hospital	104 (67.5%)	50 (32.5%)	1	1
Total hour of stay at health facilities				
12 h or less	209 (75.5%)	68 (24.5%)	1	1
13–24 h	11 (45.8%)	13 (54.2%)	0.27 (12–0.64)	0.25 (0.09–0.67)**
25 h and above	31 (72.1%)	12 (27.9%)	0.84 (41–1.72)	3.5 (0.91–13.44)
Complications during lab our and delivery				
No	229 (74.8%)	77 (25.2%)	2.16 (1.08–4.32)	2.13 (1.29–7.58)**
Yes	22 (42.1%)	16 (57.9%)	1	1

AVD, assisted vaginal delivery.

*Significant at the *p*-vale < 0.05 and **significant at the *p*-vale < 0.001.

## 4 Discussion

To recap from the above, we reiterate here that, out of the 344 respondents interviewed, we found that a full 73% of these women received friendly care, but a significant number of them (27%) had not received friendly care during childbirth. These results are consistent with the findings of a that was carried out with on postnatal mothers at the randomly selected Jimma University Specialized Hospital, the Shenen Gibe Hospital, and the Limmu Hospital; these patients had a history of ANC visits, and the provision of women-friendly care and its associated factors were assessed. Of the total respondents, 186 (71%) reported that they had received women-friendly care during ANC visits, delivery, or in postnatal wards (14).

We found that participants who had received women-friendly care had more positive childbirth experiences than did postpartum mothers, as has been reported in the literature. According to Windau Melmer, service providers must ensure that patients are as comfortable as possible and that every woman seeking care is a person of value and has the right to be treated with respect and consideration (21). Bohren et al. reported that the unfriendly care of women by health providers during childbirth was common, particularly the use of harsh or rude language (22).

This result is higher than those of 61% of the studies conducted in the Bale Zone in Southeast Ethiopia (12). This discrepancy might be due to the use of different measurement tools and study settings, the research for the Kene et al. (2022) study was carried out solely at a public hospital in Southeast Ethiopia, whereas this study was conducted across multiple centers, including hospitals and healthcare centers. Therefore, there may be differences in adherence to women-friendly care practices between hospitals and healthcare centers.

This percentage was also lower than that reported in the research at Jimma Medical center, Southwest Ethiopia (90.23%) (13). These variations may have resulted from differences in the participants' knowledge of the services provided by urban and rural residents, and different approaches to capacity building in health care training might result in different levels of women-friendly care.

Our study revealed that the primiparous participants were 2.30 times more likely to receive friendly care than women who had a parity ≥4. These findings are consistent with those of a study conducted in Southeast Ethiopia (12). A possible explanation for our findings could be that primipara mothers might consider the healthcare setting unfamiliar and believe the quality of the care provided by the facility to be perfect, while multipara mothers have had experience with the facility's care and have rated its services accordingly. The other possible reason is that multiparous women had given birth previously; thus, they were aware of what was expected of them and the healthcare providers to have a friendly relationship and receive pleasant care. However, these results are inconsistent with the findings of a related previous study (23).

The odds of receiving women friendly care were 3.02 times greater among women who had ANC follow-up than among those who did not. These findings are consistent with those of a study conducted in Harar hospitals in eastern Ethiopia (18). A possible reason may be that, when pregnancy is planned and deliberate, the mother receives continuous emotional support from her family, which has been shown to improve the quality of her childbirth experiences. The World Health Organization recommends that each pregnant woman receive her case notes during pregnancy to improve continuity, quality of care, and her pregnancy experience through ANC follow-up, which results in evidence of maternal satisfaction during childbirth (24).

This study revealed that women who gave birth during the day were 2.17 times more likely to have received women-friendly care than women who gave birth at night. These findings are in line with the results of a study conducted in Nepal (25). The reason might be that during the night, staff are more likely to experience work overload as, in a low-income country such as Ethiopia, there are generally fewer staff members lower working during the night shift.

Our also study revealed that women with a total duration of stay at healthcare facilities of 13–24 h were 75% less likely to receive women-friendly care than women with a total duration of stay of 12 h or less. These findings are similar to those of a study conducted at Nepal Medical College and Teaching Hospital (25). This similarity might be due to the correlations among the sociodemographic characteristics of the respondents. As in Nepal, the majority of respondents were housewives (63.3%). Another potential explanation is that the inadequate quality of maternal and neonatal health care in the country might lead to women with a total duration of stay at health facilities of 13–24 h being less likely to receive women-friendly care, which in turn decreases the likelihood of receiving care that is tailored to women's needs and preferences.

Additionally, our study revealed that women who did not have complications during labor or delivery were 2.13 times more likely to receive women-friendly care than women who did have had complications. These findings are consistent with those of a cross-sectional study conducted in Bahirdar, Ethiopia (26).

Our study also showed that women who gave birth at a healthcare center were 2.01 times more likely to have received women-friendly care than women who given birth at a referral hospital. This research finding aligns with those of other studies that were conducted in Ethiopia (27). Possible reasons may be that deliveries at healthcare centers involve lower rates of medication use, less use of vacuum extraction and forceps, and much lower rates of cesarean section. One potential explanation for this correlation could be the high volume of clients in hospitals, leading providers to experience busyness, burdens, and burnout (28).

### 4.1 Strength

One major advantage of this study is the minimized risk of recall bias, given that women were interviewed immediately before being discharged following childbirth. The research was conducted as part of a multicenter study encompassing both rural and urban areas. To manage potential sources of confounding, an adjusted logistic regression model was utilized.

### 4.2 Limitations

This study has notable limitations; the most efficient method for examining friendly care involved utilizing observational data collection. Additionally, while this study solely employed a quantitative approach, incorporating qualitative inquiry is crucial for obtaining comprehensive data regarding factors influencing women's access to friendly care services.

## 5 Conclusion and recommendations

In general, compared to most other findings, the level of women-friendly care provision in public health institutions in northern Ethiopia was found to be low. Variables such as place of delivery, ANC follow-up, being a primipara, complications during labour and/or birth, total hours of stay at health facilities, and daytime delivery were found to be statistically significant predictors of women-friendly care provision. We argue that strong counseling on ANC follow-up and on mothers’ stays at a healthcare facility after delivery is needed to prevent and manage complications during childbirth early to help mothers obtain friendly care.

Women-friendly care has also been included in basic and emergency obstetric care training sessions that have focused on increasing women's awareness of the importance of ANC follow-up and of potential complications during childbirth. In addition, creating awareness among women during their stays at healthcare facilities on what to expect during childbirth, including the right to informed consent and refusal, privacy, and respect for the choices and preferences during labour and delivery, help to improve women-friendly care. In addition, we strongly recommend that public healthcare institutions and other stakeholders should strengthen monitoring and evaluation mechanisms to reduce disrespectful maternal care at all times and to minimize elective inductions of labour during the night. Public health institutions should increase the number of staff who work at night, provide effective enforcement of accountability mechanisms to avoid mistreatments and support labouring and birthing women in a friendly manner.

## Data Availability

The original contributions presented in the study are included in the article/Supplementary Material, further inquiries can be directed to the corresponding author.
