# Supplementary material for: An assessment of the provision of women-friendly care and its associated factors among mothers who gave birth at public health institutions in South Gondar Zone, Northwest Ethiopia
Source: Front Glob Womens Health. 2024 May 27;5:1368388. doi: 10.3389/fgwh.2024.1368388 (PMC11163033; doi:10.3389/fgwh.2024.1368388)
Supplement: Supplementary file 1 [file Datasheet1.docx]

**Sampling technique and procedure**

A simple random sampling technique was utilized to select 1 Tertiary Hospital 2 Primary Hospitals and 20 health centers. The sample was allocated proportionally to all selected public health institutions based on the two-monthly average number of women who gave birth in each institution for the year 2020.

A systematic random sampling technique was used to select study participants from the mothers who gave birth in public health institutions of the South Gondar zone until the required sample size at each public health institution was obtained. Data were collected from every 3rd woman who gave birth during the study period at each selected health institution. The sampling interval k = 3 was calculated by dividing the source population by the total sample size and this interval was used in all health institutions to select study participants. For each of the public health institutions, the constant number K was also calculated and it was the same K=3. This interval was used in all public health institutions to select study participants. The first sample was selected randomly by lottery method among the first three participants (1 randomly selected) then every 3rd unit was taken to get the required sample size from each institution.

South Gondar zone

96 H/C and 8 hospitals

DTSH (Ni=680)

AZP/H (Ni=204)

NMP/H (Ni=160)

20 H/C (Ni=494)

154

46

36

112

348

Simple random sampling

**Proportional allocation**

Figure 2. sampling procedures to assess Women friendly care and associated factors among women who gave birth in public health institutions of south Gondar Zone, Northwest Ethiopia, 2021.

H/C=health center, DTSH=DebreTabor specialized hospital, AZ/P=adiszemen primary hospital, NMP/H=nefasmewucha primary hospital.

# Ethical considerations

The ethical clearance was obtained from the College of Medicine and Health Sciences, Debre Tabor University, then an Official formal letter of cooperation was written by the Department of Midwifery to the concerned office then a letter of support was obtained from south Gondar zone health office. Written informed consent was obtained from the individual participants. Moreover, the purpose, procedures of the study, advantages, and disadvantages were told to the participants. All the participants in the study participated voluntarily and their information was kept confidential. Participants have been informed that they have the right to withdraw any time.

Annex I: English Version Questionnaires

Part one socio-demographic characters of mother

| No. | | Question | | Response | | Remark | |
| --- | --- | --- | --- | --- | --- | --- | --- |
| 101 | | Age of mother in years | | ------------- | |  | |
| 102 | | Where is your residence? | | 1.Rural 2. Urban | |  | |
| 103 | | \| Current marital status \| \| --- \| | | 1. Single  2**.** Married  3. Divorced  4. Widowed | |  | |
| 104 | | What is your religion? | | 1. Orthodox  2. Muslim  3. Protestant  4. Catholic  5. Other (specify | |  | |
| 105 | | Ethnicity | | 1. Amhara  2. Oromo  3. Tigre  4. Other specify------- | |  | |
| 106 | | Mother’s level of education | | 1 Unable to read and write  2. Able to read and write  3. Primary (1-8) completed  4. Secondary (9-12) completed  5. Collage and above | |  | |
| 107 | | Mother’s occupation | | 1.Housewife  2. Private employee  3. Government employee  4. Merchant  5. Student  6. Other specify | |  | |
| 108 | | Families’ monthly income (in Ethiopian Birr | | ------------------------------ | |  | |
| **Part II obstetrics history of mother**  Now I am going to ask you some questions about your recent delivery to the health facility | | | | | | | |
| 201 | Current pregnancy Intended/wanted | | | | 1. Yes  2. No |  | |
| 202 | Did you have/ had ANC follow-up during this pregnancy? | | | | 1.Yes  2. No |  | |
| 203 | If you say yes in no#202, where did you receive ANC follow-up? if no, skip | | | | 1.Governmental health center  2.Governmental referral hospital  3.Governmental primary hospital  4. private clinic |  | |
| 204 | How many times did you receive ANC follow-up? In number | | | | 1. Number of times---- 2. Don’t know/ remember |  | |
| 205 | How many total numbers of deliveries you had including stillbirths/neonatal death*s* | | | | --------------------------------- |  | |
| 206 | What was the type of facility where you had your current delivery | | | | 1. Public health center  2. Public primary hospital  3. Public referral hospital |  | |
| 207 | Were there any companions (family members) during your delivery? | | | | 1. Yes 2. No |  | |
| 208 | Who was the main provider Conducting your delivery | | | | 1. Nurse  2. Midwife  3. Doctor  4. Others |  | |
| 209 | What was the sex of the main provider conducting your delivery | | | | 1. Male  2. Female |  | |
| 210 | What was the mode of your current delivery? | | | | 1. Spontaneous vaginal delivery  2. Cesarean delivery  3. Assisted vaginal Delivery |  | |
| 211 | What Is the birth outcome of this delivery? | | | | 1. Live birth 2. Stillbirth |  | |
| 212 | When is the time of delivery | | | | 1. Day time 2. Nighttime |  | |
| 213 | how many hrs did you stay in the hospital | | | | \| - - - 1. 12 h or less       2. 13 to 24 h       3. 25 h and above \| \| --- \| |  | |
| 214 | Have you had birth complications during this delivery? | | | | 1. Yes 2. No |  | |
| 215 | If you say yes for Qn #214, what happened to you? | | | | 1. Hemorrhage 2. Hypertensive disorders 3. Obstructed labor 4. Infection (postpartum) 5. Others (specify)------ |  | |
| **Part III: Categories of women received FMC** **during this childbirth in this facility** | | | | | | | |
|  | |  | Category of FMC | | | Yes | No |
| 301 | | Friendly care | I felt that health workers cared for me with a kind approach | | |  |  |
|  |  |  | The health workers treated me in a friendly manner | | |  |  |
|  |  |  | The health workers were talking positively about pain and relief | | |  |  |
|  |  |  | The health worker showed his/her concern and empathy | | |  |  |
|  |  |  | All health workers treated me with respect as an individual | | |  |  |
|  |  |  | The health worker speaks to me in a language that I can understand | | |  |  |
|  |  |  | The health provider called me by my name | | |  |  |

**የስምምነት ቅፅ**

ከላይ በተጠቀሰዉ መረጃ መሰረት በዚህ ጥናት ላይ መሳተፍ ምንም ጉዳት የለዉም።የሚሰጡት መረጃ ለማንም አይነገርም።ሰምዎትም አይጠቀስም፤መመለስ የማይፈልጉትን ጥያቄ እንዲመልሱ አይገደዱም፤በፈለጉት ጊዜ ጥያቄዉን ማቆም ይችላሉ። ለዚህ ጥናት የሚያደርጉት ትብብር ለጥናቱ መሳካት ወሳኝነዉ። በአጋጠሚ ለጥናቱ ተሳታፊ እንዲሆኑ ተመርጠዋል።

ይህ መረጃ በሚገባኝ ቋንቋ ተጠይቄ የተገለፁትን ሀሳቦች ተረድቻለሁ።

ስለዚህ በጥናቱ ለመሳተፍ ፈቃደኛነዎት?

1. ፈቃደኛ ነኝ (ቃለ ምልልሱን ይቀጥሉ)

2. ፈቃደኛ አይደለሁም( አመሰግናለሁ )

የጠያቂዉ ስምና ፊርማ____________________ቀን _______________________

የመጠይቁ ዉጤት

በሱፐርቫይዘር ተረጋግጧል

ስምና ፊርማ __________________________ ቀን_____________________

Annex II: Amharic Version Questionnaires

ክፍልአንድ: የተሳታፉውን ማንነት ዝርዝር መረጃዎች ለማወቅ የተዘጋጁ ጥያቄዎች፡

| ተ.ቁ | ጥያቄዎች | | መልስ | | ኮድ |
| --- | --- | --- | --- | --- | --- |
| 101 | የአናትየዋ እድሜ | | ----- አመት | |  |
| 102 | መኖሪያዎ የት ነው? | | 1.ገጠር 2.ከተማ | |  |
| 103 | የጋብቻ ሁኔታ | | 1. ያላገባች  2. ያገባች  3. የፈታች  4. በሞት የተለየባት  5 ሌላ ካለ ይገለፅ | |  |
| 104 | የሃይማኖት ሁኔታ | | 1. ኦርቶዶክስ 2. ሙስሊም 3. ፕሮቴስታንት 4. ካቶሊክ 5. ሌላ ካለ ይገለፅ | |  |
| 105 | የብሄር ሁኔታ | | 1. አማራ 2. ኦሮሞ 3. ትግሬ 4. ሌላከሆነ( ይገለጽ) | |  |
| 106 | የትምህርት ደረጃ ሁኔታ | | 1. አልተማረችም 2. ማንበብና መፃፍ ብቻ 3. የመጀመሪያደረጃ(1-8) ያጠናቀቀች 4. ሁለተኛደረጃ(9-12) ያጠናቀቀች 5. ኮሌጅ እና ከዚያ በላይ | |  |
| 107 | የሥራ ሁኔታ | | 1. የቤትእመቤት 2. የግልተቀጣሪ 3. የመንግስትሰራተኛ 4. ነጋዴ 5. ተማሪ 6. ሌላከሆነ( ይገለጽ) | |  |
| 108 | በወር የሚያገኙት ገቢ | | -------------- ብር | |  |
| **ክፍልሁለት፡ የእናትየዋ የወሊድ ታሪክ :** የተመረጠውን መልስ ያክብቡ | | | | | |
| ተ.ቁ | ጥያቄ | | መሌስ | | ኮዴ |
| 201 | የእርግዝና ሁኔታን የታቀደ እና የተፈለገ ነበር? | | 1.አወ  2.አደለም | |  |
| 202 | የእርግዝና ክትትል አድርገው ነበር? | | 1. አዎ 2. አላደረኩም | |  |
| 203 | የላይኛው ጥያቄ 202 አዎ ከሆነ መልስዎ የትነበር የእርግዝና ክትትል ያደረጉት? | | 1. የመንግስት ጤናጣብያ 2. የመንግስት ሪፈራል ሆስፒታል 3. የመንግስት አጠቃላይ ሆስፒታል 4. የግል ክሊኒክ | |  |
| 204 | ምን ያህል ጊዜ የእርግዝና ክትትል አድርገው ነበር? | | 1በቁጥር ይግለፁ------  2አላውቅም/አላስታዉስም | |  |
| 205 | እስከ አሁን ስንት ጊዜ ወልደው ነበር (የሞቱትን ምጨምሮ)? | | 1. አንዴ 2. ሁለት 3. ሦስት 4. አራት 5. አምስት እና ከዛ በሊይ | |  |
| 206 | አሁን የወለዱት የት ነበር? | | 1. የመንግስት ጤና ጣብያ 2. የመንግስት ሪፈራል ሆስፒታል 3. የመንግስት አጠቃላይ ሆስፒታል | |  |
| 207 | በወሊድ ወቅት የእርስዎ ጓደኞች(የቤተሰብ አባላት) ነበሩወይምገብተው ነበር? | | 1.አዎ  2. አልነበሩም | |  |
| 208 | በዋነኛነት ሲያዋልድ የነበረው ባለሙያ ማን ነበር ? | | 1. ሚድዋይፍሪ 2. ዶክተር 3. ነርስ 4. ጤና መኮንን 5. ሌላ | |  |
| 209 | በዋነኛነት ሲያዋልድ የነበረው ባለሙያ ፆታ ? | | 1. ወንድ 2. ሴት | |  |
| 210 | በምን አይነት ሁኔታ ነበር የወለዱት? | | 1. በማህፀን 2. በቀዶ ጥገና 3. በመሳሪያ ድጋፍ | |  |
| 211 | እንደተወለደ የልጅዎ ሁኔታ እንደት ነበር? | | 1.ጥሩ ነበር  2.ሞቶ ነዉ የተወለደዉ | |  |
| 212 | የወለድሽበት ስአት መች ነበር? | | 1.ቀን ስአት  2.ማታ ስአት | |  |
| 213 | ከወለዱ በኀላ ሆስፒታል ቆይተዋል? | | 1.አዎ 2.አይደለም | |  |
| 214 | ለላይኛው ጥያቄ ቁ213 አዎከሆነ መልስዎ፡፡ስንት ቀን ቆዩ? | | 1. ከ 12 ስአት ያነሰ 2. ከ 13 ስአት እስከ 24 ስአት 3. ከ 25 ስአት በላይ | |  |
| 215 | በወሊድ ወቅት ችግር አጋጥሞዎት ነበር? | | 1. አዎ 2.አይደለም | |  |
| 216 | ለላይኛው ጥያቄ  ቁ215 አዎ ከሆነ መልስዎ ምን አይነት ችግር ነበር ያጋጠመዎት? | | 1. የደም መፉሰስ 2. የደም ግፊት 3. ወሊድ እንፌክሽን 4. አስቸጋሪ ምጥ 5. ሌላ ከሆነ ይጥቀሱ | |  |
| **ክፍል ሦስት: በጤና ተቋም ሲወሊድ** ቀረቤታ ያለው እንክብካቤ በተመለከተ | | | | | |
| ተ.ቁ |  | የአገልግሎቱ አይነት | | አወ | አይደለም |
| 301 | ቀረቤታ ያለው እንክብካቤ በተመለከተ | 1.ባለሙያው አገልግሎት የሰጠሽ በጸባይ ነው? | |  |  |
|  |  | 2.ባለሙያዎቹ የተንከባከቡሽ ቀረቤታ በተሞላበት ነው | |  |  |
|  |  | 3.የጤና ሰራተኞች ስለ ህመምሽና ስለመዳንሽ የነገሩሽ በአዎንታዊ  መንገድ ነው? | |  |  |
|  |  | 4.የጤና ባለሙያው ችግርሽን እንደራሱ ችግር  እና በርህራሄ አይቶት ነበር ? | |  |  |
|  |  | 5.የጤና ባለሙያዎቹ የተንከባከቡሽ በአክብሮት እንደማንኛውም ሰው ነው? | |  |  |
|  |  | 6.የጤና ባለሙያዎቹ ያናገሩሽ በሚገባሽ ቋንቋነው ? | |  |  |
|  |  | 7.የጤና ባለሙያዎቹ የሚጠሩሽ በስምሽ ነው | |  |  |
